# Supplementary material for: Optimizing large language models for detecting symptoms of depression/anxiety in chronic diseases patient communications
Source: NPJ Digit Med. 2025 Sep 30;8:580. doi: 10.1038/s41746-025-01969-5 (PMC12485036; doi:10.1038/s41746-025-01969-5)
Supplement: Supplementary file 1 — npjDM_MH_LLM_Rev_Supplement_Sub [file 41746_2025_1969_MOESM1_ESM.pdf]

Optimizing Large Language Models for Detecting Symptoms of Depression/Anxiety in Chronic Diseases Patient Communications

Supplementary documents

Supplementary Note 1. Depression or anxiety-related keywords
Supplementary Note 2. Prompts for zero-shot, few-shot, systemic persona, and PHQ-4 classification
Supplementary Note 3. Engineered prompts for the systemic persona

Supplementary Data 1. LLMs' evaluations: Challenging cases → As a separate table
Supplementary Data 2. LLMs' evaluations: zero-shot and few-shots → As a separate table
Supplementary Data 3. LLMs' evaluations: with vs without persona → As a separate table
Supplementary Table 1. Cost and inference speed of the models
Supplementary Table 2. Sociodemographic characteristics of patients

Supplementary Note 1. Depression or anxiety-related keywords

First, we transformed preprocessed raw data into sentences using a pre-calculated embedding model (all-miniLM-L6-v2) that turns texts into a 384-vector, extensively pre-trained BERT-like transformer with over 600 million social media posts and 12 million medical journals[1]. We applied Uniform Mapping and Approximation and Projection (UMAP) to reduce the dimensionality of embeddings and ConvectVectorizer to delete uncommon words. To create key topics clustered based on similarity score, we leveraged an unsupervised zero-shot approach, setting the similarity threshold as 0.82. To hone the initial topic clustering and improve the interpretability of the topics, we built a second topic model. We leveraged the Balanced Iterative Reducing and Clustering (BIRCH) algorithm as the BIRCH algorithm can effectively and efficiently handle large data through both hierarchical and partitioning strategies[2]. The second topic model creased a list of key topics with representative keywords for each topic. We obtained keywords grouped with depression or anxiety, like below. Code for topic modeling is available at GitHub (https://github.com/JK0902/MH_LLM)

1. Keywords that we identified using NLP topic modeling to obtain patients' own language to describe their symptoms

**['anxiety', 'anxiousness', 'anxious', 'panic', 'nervousness', 'symptom', 'stress', 'worry', 'depression', 'experiencing']**
**['depression', 'depressive', 'depressed', 'stressed', 'psychiatry', 'mood', 'stress', 'anxiety', 'disorder', 'misery']**

2. Keywords that we empirically identified which frequently appeared together with "depressed/depressive/depression" or "anxious/anxiety" in the samples that we screened: *tired, exhausted*

References

1. sentence-transformers/all-MiniLM-L6-v2 · Hugging Face. January 18, 2024. Accessed May 3, 2024. https://huggingface.co/sentence-transformers/all-MiniLM-L6-v2

2. Zhang T, Ramakrishnan R, Livny M. BIRCH: A New Data Clustering Algorithm and Its Applications. *Data Min Knowl Discov*. 1997;1(2):141-182. doi:10.1023/A:1009783824328

Supplementary Note 2. Prompts for zero-shot, few-shot, systemic persona, and PHQ-4 classification

1. All the code used to assess LLMs through API (Llama 3.1 8B instruct, Llama 3.1 405B instruct, Gemini Pro 1.5) is available at GitHub (https://github.com/JK0902/MH_LLM)

2. Prompts used for OpenAI o1 and DeepSeek R1 assessed in Stanford Secure GPT environment are below.

- Binary classification with zero-shot:

```
full_prompt = (
            f"Classify each message as 1 if the person seems to have
depression or anxiety, otherwise 0.\n"
            f"Output format:\n"
            f"Classification: <0 or 1>\n"
        )
; message:
```

- Binary classification with few-shot (n=2):

```
reference = (
"Example 1:\n"
    "Patient query: xyz\n"
    "Clinician's decision: Yes (1), depression/anxiety.\n"
    "Clinician's reasoning: The message describes a change in work
function due to anxiety (not been able to go to work)."
    "The individual also is not eating or sleeping well, both are signs of
depression. The individual describes not feeling safe driving,"
    "and not being himself. According to the Centers for Disease
Prevention (CDC) and research studies,"
    "there are several important risk factors for suicide risk, including
increased anxiety, sleeping too little or too much,"
    "which are included in this message (https://www.cdc.gov/suicide/risk-
factors/index.html). I would call them on the phone,"
    "and further assessment for anxiety, depression and suicide risk,
giving hotline for suicide 988 and referral for psychiatry evaluation if
needed.\n"

"Example 2:\n"
    "Patient query: abc\n"
    "Clinician's decision: No (0), depression/anxiety.\n"
    "Clinician's Reasoning: Rash that covers the whole body and is itchy
is a sign of an allergic reaction. "
```

```
    "In this context, feeling fatigue and run down is a result of medical
illness. "
    "Fatigue (although a symptom of depression) can be attributed to
medical condition; according to DSM-5, "
    "a mental health diagnosis like depression can be made when the
symptoms are not attributed to a medical condition.\n"
)
        full_prompt = (
            f"Learn from: {reference}\n"
            f"Classify each message as 1 if the person seems to have
depression or anxiety, otherwise 0.\n"
            f"Output format:\n"
            f"Classification: <0 or 1>\n"
        )
; message:
```

- PHQ-4 classification (base)

```
criteria = (
    "little interest (little interest or pleasure in doing things), "
    "hopeless (feeling down, depressed, or hopeless), "
    "nervous (feeling nervous, anxious or on edge), "
    "worrying (not being able to stop or control worrying)"
)
question = "What is the likelihood of little interest, hopeless, nervous,
and worrying for each message?"
requirements = (
    "Please provide the likelihood for each criterion on a 4-point Likert
scale "
    "(0=not likely, 1=unlikely, 2=likely, 3=very likely) and sum all the
Likert scales for each message."
)
prompt_template = (
    f"Answer this question: {question} using {criteria} "
    f"and follow the requirements: {requirements}\n"
    f"Respond in the following format strictly:\n"
    f"1. Little interest: <0-3>\n"
    f"2. Hopeless: <0-3>\n"
    f"3. Nervous: <0-3>\n"
    f"4. Worrying: <0-3>\n"
    f"Sum of Likert scales: <sum>"
); message:
```

- PHQ-4 classification with systemic persona

```
system_instruction =(
    "As Dr. GPT, a professional psychiatrist in the hospital,"
    "one of your roles is responding to the patients messages through the patient portal."
    "Patients ask health questions and report some symptoms or lab results."
    "I will ask for you to evaluate the patients messages to screen those who would need further assessment for depression or anxiety."
    "I myself am a psychiatrist in the hospital. I will use your evaluation to better detect"
    "patients depressive and anxious symptoms timely through the patient portal messages."
    "For each question, you should take time to think deeply and step-by-step to be sure to offer"
    "the right answer in a professional manner. To begin, please confirm that you understand your role."
)

criteria = (
    "little interest (little interest or pleasure in doing things), "
    "hopeless (feeling down, depressed, or hopeless), "
    "nervous (feeling nervous, anxious or on edge), "
    "worrying (not being able to stop or control worrying)"
)
question = "What is the likelihood of little interest, hopeless, nervous, and worrying for each message?"
requirements = (
    "Please provide the likelihood for each criterion on a 4-point Likert scale "
    "(0=not likely, 1=unlikely, 2=likely, 3=very likely) and sum all the Likert scales for each message."
)
prompt_template = (
    f"{system_instruction}\n"
    f"Answer this question: {question} using {criteria} "
    f"and follow the requirements: {requirements}\n"
    f"Respond in the following format strictly:\n"
    f"1. Little interest: <0-3>\n"
    f"2. Hopeless: <0-3>\n"
    f"3. Nervous: <0-3>\n"
    f"4. Worrying: <0-3>\n"
    f"Sum of Likert scales: <sum>"
); message:
```

Supplementary Note 3. Engineered prompts for the systemic persona

Researcher *[provided context using multiple techniques, including role prompting, directive commanding, expertise emulation, and zero-shot chain of thought]:*

As, Dr. GPT, a professional psychiatrist in the hospital, one of your roles is responding to the patients messages through the patient portal. Patients ask health questions and report some symptoms or lab results. I will ask for you to evaluate the patients messages to screen those who would need further assessment for depression or anxiety.

I myself am a psychiatrist in the hospital. I will use your evaluation to better detect patients depressive and anxious symptoms timely through the patient portal messages.

For each question, you should take time to think deeply and step-by-step to be sure to offer the right answer in a professional manner. To begin, please confirm that you understand your role.

Supplementary Table 1. Cost and inference speed of the models

**Model**	**Inference Speed**	**Cost (Per million tokens)**	
	(Per second)	Input Tokens	Output Tokens
Llama 3.1 8B (Instruct)	226.1 tokens	N/A	N/A
Llama 3.1 405B (Instruct)	969 output tokens	$5.00	$16.00
Gemini Pro 1.5	N/A	$0.00125/1k characters	
Gemini Pro 2.0 (Flash Lite)	6720 tokens (Per-second throughput per GSU)	$0.0075	$0.30
OpenAI o1	143 tokens	$16.50 (01 2024-12-17-Regional)	$66 (01 2024-12-17-Regional)
OpenAI o3-mini	180.3 tokens	$1.21 (03-mini 2025-01-31 Regional)	$4.84 (03-mini 2025-01-31 Regional)
DeekSeek R1	250 tokens	$2.36	

a. Llama 3.1 models and Gemini models were used through Vertex AI platform, a HIPPA-compliant environment (Google LLC).
b. The Llama 3.1 API service was at no cost during public preview (available at no charge until May 1, 2025)
c. In our study, due to the private nature of patient message data, OpenAI and DeekSeek R1 models were analyzed in a secure institutional platform at no charge. In this Table, we listed the currently available pricing information through Microsoft Azure platform.
d. We did not evaluate inference speed at the time of study. Inference speed could vary by setting, and the information in this Table could be used as approximate estimation.

Supplementary Table 2. Sociodemographic characteristics of patients

	**Patients with CVD and co-morbid dep/anx (N=303)[a]**		**Patients with CVD and without co-morbid dep/anx (N=292)[a]**	
	Frequency (N)	(%)	Frequency (N)	(%)
**Age**				
18-34	8	2.6	7	2.4
35-49	33	10.9	23	7.9
50-64	119	39.3	75	25.7
65-74	51	16.8	103	35.3
75+	92	30.4	84	28.8
**Race**				
Asian/Pacific Islander	59	19.5	73	25.0
Black	10	3.3	15	5.1
Native American/Other	37	12.2	41	14.03
White	196	64.7	157	53.8
Unknown	1	0.3	6	2.1
**Ethnicity**				
Hispanic/Latino	27	8.9	34	11.6
Non-Hispanic	264	87.1	245	83.9
Unknown	12	4.0	13	4.5
**Sex[c]**				
Female	206	68.0	162	55.5
Male	97	32.0	130	44.5

a. A total number of messages are 606 (n=306 for those with dep/anx, n=300 for those without dep/anx). While we randomly selected the messages, some messages from the same individuals were included; Abbreviations: dep (Depression), anx (Anxiety).
